# Family History of Hypertension and Echocardiographic Left Ventricular Hypertrophy in Hypertensive Nigerians

**DOI:** 10.1155/2024/7858899

**Published:** 2024-09-21

**Authors:** Olugbenga Olusola Abiodun, Tina Anya, Victor Tunde Adekanmbi, Dike Ojji

**Affiliations:** ^1^ Department of Internal Medicine Federal Medical Centre, Abuja, Nigeria; ^2^ Department of Obstetrics and Gynecology University of Texas Medical Branch at Galveston, Galveston, Texas, USA; ^3^ Department of Internal Medicine University of Abuja University of Abuja Teaching Hospital, Gwagwalada, Abuja, Nigeria

## Abstract

**Introduction:**

Studies on the relationship between a family history of hypertension and left ventricular hypertrophy are sparse. We evaluated this relationship in patients with essential hypertension.

**Methods:**

A total of 1668 patients with essential hypertension were consecutively enrolled in the prospective Federal Medical Centre Abuja Hypertension Registry. First-degree family history was defined by the presence of a known history of hypertension in any or both parents, siblings, and children. Echocardiographic left ventricular hypertrophy was diagnosed using the criteria of the American Society of Echocardiography and the European Association of Cardiovascular Imaging.

**Results:**

The prevalence of a family history of hypertension, echocardiographic, and electrocardiographic left ventricular hypertrophy were 61.7%, 46.8%, and 30.8%, respectively. After multivariable adjustment, paternal history of hypertension [OR: 1.56, CI: 1.20–2.05, *p*=0.001] was associated with an increased risk of echocardiographic left ventricular hypertrophy, while maternal history of hypertension [OR: 0.72, CI 0.58–0.91, *p*=0.006] was associated with a reduced risk. Age ≥50 years (*p*=0.026), duration of hypertension ≥1 year (*p*=0.047), and heart failure (*p* < 0.001) were associated with an increased risk of left ventricular hypertrophy, while male sex (*p* < 0.001) was associated with a reduced risk.

**Conclusion:**

Our study showed that a paternal history of hypertension is associated with an increased left ventricular hypertrophy risk among patients with essential hypertension, while maternal history is protective.

## 1. Introduction

Left ventricular hypertrophy (LVH) defined as an increase in the left ventricular mass index (LVMI) increases cardiovascular (CV) risk in hypertensive and normotensive individuals [1–3]. The prevalence of LVH is high among patients with hypertension, and it is often severe [4]. After 36 years of follow-up in the Framingham Heart Study, the risk ratios of electrocardiographic LVH (ECG-LVH) were greatest for heart failure (HF), stroke, and coronary artery disease (CAD), and LVH was found to be an important contributing mechanism for sudden cardiac death in hypertensive patients [5]. Whether diagnosed by electrocardiography (ECG) or echocardiography, there is ample evidence that LVH is a potent independent predictor of morbidity and mortality irrespective of blood pressure (BP) [1, 6].

Family-based studies have shown that ECG and echocardiography-related LVH are heritable [7–10]. According to the Dutch Hypertension and Offspring study, a family history of hypertension was associated with an increase in the LVMI in children of two parents with hypertension [11]. Additionally, a study of normotensive African American and Caucasian youths showed increased LVM/body surface area among those with a family history of essential hypertension [12]. These data have been extrapolated to hypertensive patients because data on the influence of family history on LVH in hypertensive patients are sparse.

We hypothesized that a positive family history of hypertension is associated with echocardiographic LVH in patients treated for hypertension. This study investigated whether a first-degree family history of hypertension in patients with essential hypertension confers an increased risk of developing LVH compared to patients without a family history of essential hypertension. This association has the possibility of causing early use of electrocardiography in communities or primary care clinics in Nigeria and sub-Saharan Africa. This could lead to early referral for echocardiography to identify those with LVH and prompt intervention to reduce sudden death, CAD, HF, chronic kidney disease (CKD), and stroke.

## 2. Methods

### 2.1. Subjects

One thousand six hundred and sixty-eight (1,668) essential hypertensive patients aged 18 years and older with complete data from the prospective Federal Medical Centre Abuja Hypertension Registry (FMCAHR) were analyzed for this study. All patients with secondary forms of hypertension, including pregnancy-related hypertension and those whose family history of hypertension was not known, were excluded from this analysis.

**Figure 1:**
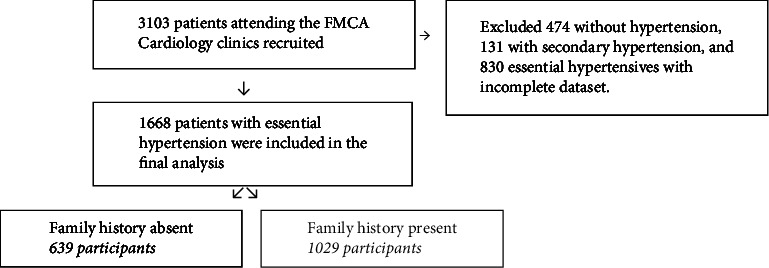
Data profile. FMCA: Federal Medical Centre, Abuja.

All consenting patients attending the Cardiology Clinics of the Federal Medical Centre, Abuja (FMCA), were consecutively recruited into the FMCAHR from 2016 to 2021. 3,103 patients were enrolled consecutively after providing informed consent and were followed up (Figure 1). Ethical approval was obtained from the hospital's ethics research committee under registration number FMCABJ/HREC/2017/009.

### 2.2. Clinical Information

The data of patients stored in the Excel database included age (years), sex, weight (kilograms), height (centimetres), body mass index (BMI) (kilograms/meter^2^), waist circumference (centimetres), duration of hypertension and diabetes mellitus (DM) (in weeks, months, and years), first-degree family history (parents, father, mother, siblings and children) of hypertension and DM, intake of alcohol in grams and duration, use of tobacco and number of pack years, recreational drug use, BP (mmHg) measurements taken using a mercury sphygmomanometer (Accosson, London, UK), presence of CV complications, and antihypertensive medications.

### 2.3. Laboratory Evaluation

Blood was drawn at the hospital laboratory during the first visit for fasting blood glucose (FBG), fasting lipid profile (FLP), and electrolyte, urea, and creatinine (EUCr) levels. Follow-up repeats of these tests were performed as needed. The estimated glomerular filtration rate (eGFR) was calculated using the 2021 Chronic Kidney Disease Epidemiology Collaboration Creatinine Equation (CKD-EPI) [13]. Resting 12-lead electrocardiography (ECG) was performed on all patients using the Schiller AT-102 system with serial number 070.07911 (Switzerland) at a speed of 25 mm/s and 1 mV/cm calibration. The ECGs were reported by the authors.

### 2.4. Echocardiography

Echocardiography was performed and reported by the first two authors who are experienced cardiologists. Echocardiography was performed at the patient's first visit using General Electric, Model Vivid E9, GA314809-03 (Norway) or General Electric, Model S6, 050140VS6N (Norway). Measurements were taken using the recommendations of the American Society of Echocardiography and the European Association of Cardiovascular Imaging [14, 15].

### 2.5. Definitions

- LVH was defined as an LVMI >95 g/m2 in females and >115 g/m2 in males according to the American Society of Echocardiography [15]. LVMI was calculated using the linear cube formula of the American College of Cardiology: 0.8 (1.04 ([LVIDD + PWTD + IVSTD]^3^ − [LVIDD]^3^)) + 0.6 g and indexing with body surface area [1, 14, 15]. Relative wall thickness (RWT) was calculated with the formula: 2 × posterior wall thickness/LV internal diameter at end-diastole [1].
- First-degree family history was defined by the presence of a known history of hypertension in any or both parents, siblings, and children, that is, individuals who share 50% of the patients' genes [16].
- Hypertension was diagnosed by persistent elevation of office BP ≥140/90 mmHg [1], use of antihypertensive medication, or prior diagnosis by a physician before referral to our clinic.
- DM was diagnosed according to the American Diabetes Association as two abnormal blood glucose readings of FBG >7.0 mmol/l (126 mg/dl) or random blood glucose (RBG) > 11.1 mmol/l (180 mg/dl), and glycated hemoglobin (HbA1C) > 6.5% [17], use of antidiabetic medications, or prior diagnosis by a physician before referral to our clinic.
- Dyslipidemia was defined as the presence of any change in lipids (lipoproteins), whether isolated or combined [18].
- HF was defined as the presence of symptoms and signs with echocardiographic evidence of structural heart disease and or elevated filling pressures [19].
- CAD, HF, CKD, and cerebrovascular disease (CVD) were diagnosed by the authors and other hospital physicians according to guideline recommendations [1, 20].
- Cardiovascular complications were defined as the presence of abnormal LV geometry and complications such as HF, CAD, CVD, and CKD present at the first visit or during follow-up [1, 20].
- Electrocardiographic LVH (ECG-LVH) was defined as the presence of any of Sokolow–Lyon, Cornell voltage, and Gubner–Ungerleider criteria [1].

### 2.6. Data Analysis

Categorical variables are expressed as proportions and percentages, while continuous variables are expressed as the means ± standard deviations or as ranges. The associations between the characteristics of participants with a family history of hypertension and echocardiographic LVH were tested by chi-square tests and independent *t*-tests. Fisher's exact test was used for categorical data with an expected cell size of less than 5. Multivariate logistic regression with model-fitting statistics was used to determine the relationships between the significant univariate models and echocardiographic LVH. The data were analyzed using SPSS version 26 software for Windows. A *p* value <0.05 was considered to indicate statistical significance.

## 3. Results

### 3.1. Participant Characteristics by Family History of Hypertension

The baseline characteristics of the participants by family history of hypertension are presented in Table 1. The prevalence of a positive family history of hypertension was 61.7%. The mean total age of the participants was 55 ± 13 years, and the participants were predominantly females (55.8%).

Participants with a family history of hypertension were significantly younger (*p* < 0.001) than those without a family history of hypertension and those with a family history of hypertension had a higher BMI (*p* < 0.001). Those with a family history of hypertension had more cardiovascular complications (*p*=0.039) and a longer mean duration of hypertension (*p* < 0.001) than those without. Additionally, the eGFR (*p*=0.008), mean number of antihypertensive medications (*p*=0.048), use of calcium channel blockers (*p* < 0.001), and arterial vasodilators (*p*=0.037) were greater in the group with a family history of hypertension. The prevalence of ECG LVH, echocardiographic LVH, alcohol, and tobacco use among all participants was 30.8%, 46.8%, 31.7%, and 6%, respectively. Office SBP and DBP, ECG LVH, RWT, LVMI, FBG, creatinine, and all cholesterol were similar between those with a family history of hypertension and those without. Additionally, the use of diuretics, angiotensin-converting enzyme inhibitors (ACEIs), angiotensin receptor blockers (ARBs), beta-blockers, alpha-blockers, and centrally acting agents showed no difference between the two groups (*p* > 0.05).

### 3.2. Associations with Echocardiographic LVH

Table 2 shows the univariable analysis of factors associated with echocardiographic LVH. The prevalence of echocardiographic LVH in this population of hypertensive patients was 46.8%. The table also shows that there was no association between family history of hypertension (*p*=0.904), parental history (*p*=0.128), sibling history (*p*=0.125), and echocardiographic LVH. However, maternal (*p* < 0.001) and paternal (*p*=0.030) histories of hypertension were significantly associated with echocardiographic LVH. In addition, age (*p*=0.001), sex (*p*=0.006), duration of hypertension (*p*=0.006), HF (*p* < 0.001), and CKD (*p*=0.006) were significantly associated with echocardiographic LVH. However, BP level, DM, and obesity did not significantly differ.

### 3.3. Multivariate Logistic Regression Analysis

Table 3 shows that after multivariable adjustment, the odds of echocardiographic LVH increased in patients aged ≥50 years (*p*=0.026), with a duration of hypertension ≥1 year (*p*=0.047), with a paternal history of hypertension (*p*=0.001), and with the presence of HF (*p* < 0.001). However, the odds of echocardiographic LVH were lower in males (*p* < 0.001) and those with a maternal history of hypertension (*p*=0.006).

## 4. Discussion

To the best of our knowledge, the relationship between a family history of hypertension and echocardiographic LVH in patients with essential hypertension has not been explored. This study showed the prevalence of a family history of hypertension of 61.7%. Additionally, our study showed that hypertensive patients with a paternal history of hypertension were 1.56 times more likely to have echocardiographic LVH, while those with a maternal history were less likely to have LVH. In this study, the prevalence of LVH diagnosed by echocardiography was 46.8%.

There is a paucity of data on family history of hypertension in Nigeria and Africa. According to a population-based cross-sectional survey of 6,102 Caucasian participants in Switzerland, the prevalence of a family history of hypertension was 39.6% [21]. This finding contrasts with our study, possibly because our study was hospital-based, and we used a cohort of hypertensive patients and not a general population cohort. Our study showed a strong positive correlation between family history and hypertension, with more than 60% of our participants having a family history of hypertension in first-degree relatives. Taken together, these findings suggest that genetics, epigenetics, and shared environmental factors are possibly important mechanistic elements in the aetiopathogenesis of essential hypertension and that integrating family counselling into lifestyle interventions for hypertension may be an effective preventive tool to improve hypertension awareness in Nigerian communities.

Participants with a paternal history of hypertension had a 56% increased risk of developing LVH, while those with a maternal history had an 18% reduced risk of LVH. Few studies performed on family history and echocardiographic LVH have been in children and young people. In the Dutch Hypertension and Offspring study [11], hemodynamic parameters on 24-hour ambulatory BP (24-h ABPM) and LVMI were studied in three groups of normotensive children with different familial dispositions for hypertension. They found an increase in the LVMI in the offspring of two hypertensive parents compared to the LVMI in the offspring of two normotensive parents and one hypertensive parent. In a study of 323 African American and Caucasian normotensive youths, a family history of essential hypertension was associated with increased LVM/BSA [10]. Kolo et al., in a Nigerian study of 65 offspring of hypertensive parents, showed that the mean LVMI was greater in the offspring of hypertensive parents than in their age and sex-matched controls despite having similar BPs [22]. However, none of these studies examined the impact of paternal or maternal history on the LVMI. According to our study, a paternal history of hypertension is a risk factor for the development of LVH, while a maternal history is a protective factor. This finding is similar to epidemiological studies that have suggested increased adverse outcomes among offspring of fathers with metabolic diseases [23, 24]. Paternally inherited genetic mutations and epigenetic reprogramming of male germ cells have been proposed as possible mechanisms for this increased paternal disease risk [23–26]. Further studies are needed to confirm our findings and if confirmed, further genetic and epigenetic studies may provide insights into the factors and functional genomic regions underpinning cardiac hypertrophy. This may impact public health through the development of preventive strategies that can reduce the CV risk of LVH.

In this study, the prevalence of LVH diagnosed by echocardiography was 46.8%, which falls within the range of what has been found in previous studies. The prevalence of echocardiographic LVH in the literature varies widely from 3% to 77% depending on the study population and patient characteristics [4, 27, 28]. According to clinical practice studies of Nigerian hypertensive individuals, the prevalence of echocardiographic LVH varies from 30.9% to 56% [29, 30], while in an Italian nationwide multicenter survey of 2249 patients, the prevalence was 58% [4]. In a review of echocardiographic LVH of a pooled population of 37,700 hypertensive patients in 30 studies, most of whom were Caucasian, the prevalence ranged from 36% to 46.2% [27]. Our study showed that LVH is a common finding in patients with hypertension; therefore, efforts at aggressive management of hypertension and preventive measures against CV risks of hypertension and LVH should be prioritized.

One of the strengths of this study lies in the relatively large population studied. Additionally, to our knowledge, the relationship between family history and LVH has not been previously studied among patients with hypertension. However, this was a practice-based study, and the results may be different if the study was performed in the community. Therefore, there is a need to replicate this study in a large and diverse population-based study. These findings will validate our findings, expand the applicability of the findings, and hopefully form the basis for future research on the genetic epidemiology of hypertension and hypertensive LVH.

## 5. Conclusion

The findings of this study of hypertensive Nigerians showed that paternal history of hypertension significantly predicts echocardiographic LVH, and maternal history is protective. A high prevalence of a family history of hypertension was also revealed. Confirmatory studies to ascertain the influence of paternal inheritance of hypertension on LVH are important for studying the genetic and epigenetic mechanisms contributing to the development of LVH in patients with essential hypertension and their exact roles. Our study showed that LVH is common and is observed in nearly 1 out of every 2 Nigerian hypertensive patients in the hospital setting. This finding suggests an increased risk of cardiovascular morbidity and mortality among Nigerian hypertensive patients and calls for early screening of hypertension for echocardiographic LVH. Since Nigeria is largely rural, community use of ECGs with the referral of hypertensive patients with ECG LVH/abnormalities and a paternal family history of hypertension for echocardiography may aid the prompt diagnosis of LVH. Early use of medications that reverse LVH in this group of patients will reduce CV morbidity and mortality.

## Figures and Tables

**Table 1:** Baseline characteristics of participants by family history of hypertension.

Variables	Total	Family history absent	Family history present	*P* value
	*n* = 1668	*n* = 639 (38.3%)	*n* = 1029 (61.7%)	
Age (years)	55 ± 13	57.0 ± 13.2	53.46 ± 12.5	<0.001
Sex				
Male	737 (44.2%)	305 (47.7%)	432 (42.0%)	0.022
Female	931 (55.8%)	334 (52.3%)	597 (58.0%)	
BMI (kg/m^2^)	30.6 ± 6.0	29.7 ± 6.0	31.1 ± 5.9	<0.001
Duration of HTN (years)	8.2 ± 8.3	6.8 ± 7.7	9.0 ± 8.5	<0.001
Diabetes mellitus	330 (19.8%)	133 (20.9%)	197 (19.2%)	0.404
Alcohol intake				
No	1114 (66.8%)	441 (69.0%)	673 (65.4%)	0.128
Yes	554 (33.2%)	198 (31.0%)	356 (34.6%)	
Tobacco intake				
No	1564 (93.8%)	603 (94.4%)	961 (93.5%)	0.466
Yes	103 (6.2%)	36 (5.6%)	67 (6.5%)	
First office SBP (mmHg)	147 ± 23	147 ± 23	147 ± 22	0.641
First office DBP (mmHg)	90 ± 26	89 ± 14	90 ± 32	0.264
ECG LVH	497 (30.8%)	185 (29.9%)	312 (31.4)	0.539
Relative wall thickness	0.49 ± 0.1	0.49 ± 0.1	0.50 ± 0.1	0.555
LVMI (g/m^2^)	109.7 ± 37.5	110.1 ± 40.2	107.9 ± 35.8	0.238
FBG (mmol/l)	6.0 ± 2.4	6.1 ± 2.7	6.0 ± 2.3	0.483
Creatinine (umol/l)	97.5 ± 35.7	99.3 ± 31.7	96.3 ± 37.9	0.103
eGFR (ml/min per 1.73 m^2^)	73.0 ± 20.0	71.3 ± 20.3	74.0 ± 19.4	0.008
Total cholesterol (mmol/l)	5.1 ± 1.3	5.1 ± 1.3	5.1 ± 1.3	0.633
HDL cholesterol (mmol/l)	1.6 ± 0.6	1.6 ± 0.6	1.6 ± 0.6	0.105
LDL cholesterol (mmol/l)	3.1 ± 1.1	3.1 ± 1.1	3.1 ± 1.1	0.874
Triglycerides (mmol/l)	1.2 ± 0.6	1.2 ± 0.6	1.2 ± 0.6	0.157
Number of antihypertensive agents	2.6 ± 1.2	2.6 ± 1.1	2.7 ± 1.2	0.048
Diuretics	888 (53.2%)	341 (53.4%)	547 (53.2%)	0.935
Calcium channel blockers	1146 (68.7%)	406 (63.5%)	740 (71.9%)	<0.001
ACEIs	680 (40.8%)	275 (43.0%)	405 (39.4%)	0.137
ARBs	644 (38.6%)	242 (37.9%)	402 (39.1%)	0.525
Beta‐blockers	578 (34.7%)	215 (33.6%)	363 (35.3%)	0.496
Alpha‐blockers	67 (4.0%)	25 (3.9%)	42 (4.1%)	0.864
Arterial vasodilators	7 (0.4%)	0 (0.0%)	7 (0.7%)	0.037
Centrally acting	65 (3.9%)	18 (2.8%)	47 (4.6%)	0.072
Cardiovascular complications				
No	463 (27.8%)	159 (24.9%)	304 (29.5%)	0.039
Yes	1205 (72.2%)	480 (75.1%)	725 (70.5%)	

ACEIs: angiotensin‐converting enzyme inhibitors, ARBs: angiotensin receptor blockers, BMI: body mass index, DBP: diastolic blood pressure, ECG: electrocardiographic, eGFR: estimated glomerular filtration rate, FBG: fasting blood glucose, HTN: hypertension, LVH: left ventricular hypertrophy, LVMI: left ventricular mass index, SBP: systolic blood pressure.

**Table 2:** Univariable analysis of factors associated with echocardiographic LVH.

Variables	Total *n* = 1668	ECHO LVH absent *n* = 887 (53.2%)	ECHO LVH present *n* = 781 (46.8%)	*P* value
Age				
<50 years	562 (33.8%)	330 (37.3%)	232 (29.8%)	0.001
≥50 years	1101 (66.2%)	554 (62.7%)	547 (70.2%)	
Sex				
Male	737 (44.2%)	420 (47.4%)	317 (40.6%)	0.006
Female	931 (55.8%)	467 (52.6%)	464 (59.4%)	
Duration of HTN				
<1 year	350 (21.0%)	209 (23.6%)	141 (18.1%)	0.006
≥1 year	1318 (79.0%)	678 (76.4%)	640 (81.9%)	
Blood pressure level				
<140/90 (mmHg)	384 (23.0%)	220 (24.8%)	164 (21.1%)	0.070
≥140/90 (mmHg)	1282 (77.0%)	667 (75.2%)	615 (78.9%)	
Diabetes mellitus				
Absent	1333 (80.2%)	719 (81.2%)	614 (78.9%)	0.236
Present	330 (19.8%)	166 (18.8%)	164 (21.1%)	
Obesity				
Absent	837 (50.4%)	450 (51.0%)	387 (49.7%)	0.585
Present	824 (49.6%)	432 (49.0%)	392 (50.3%)	
Family history of HTN				
Absent	639 (38.3%)	341 (38.4%)	298 (38.2%)	0.904
Present	1029 (61.7%)	546 (61.6%)	483 (61.8%)	
Parental history of HTN				
Absent	1317 (79.0%)	713 (80.4%)	604 (77.3%)	0.128
Present	351 (21.0%)	174 (19.6%)	177 (22.7%)	
Maternal history of HTN				
Absent	1183 (70.9%)	596 (67.2%)	587 (75.2%)	<0.001
Present	485 (29.1%)	291 (32.8%)	194 (24.8%)	
Paternal history of HTN				
Absent	1377 (82.6%)	749 (84.4%)	628 (80.4%)	0.030
Present	291 (17.4%)	138 (15.6%)	153 (19.6%)	
Sibling history of HTN				
Absent	1261 (75.6%)	684 (77.1%)	577 (73.9%)	0.125
Present	407 (24.4%)	203 (22.9%)	204 (26.1%)	
Heart failure				
Absent	1445 (86.6%)	859 (96.8%)	586 (75.0%)	<0.001
Present	223 (13.4%)	28 (3.2%)	195 (25.0%)	
Chronic kidney disease				
Absent	1632 (97.8%)	876 (98.8%)	756 (96.8%)	0.006
Present	36 (2.2%)	11 (1.2%)	25 (3.2%)	

ECHO: echocardiography, HTN: hypertension, LVH: left ventricular hypertrophy.

**Table 3:** Multivariate logistic regression analysis of factors associated with echocardiographic LVH.

Outcome (echocardiographic LVH)		
Covariates	Adjusted odds ratio (95% CI)	*P* value
Age ≥50 years	1.29 (1.03–1.62)	0.026
Male sex	0.68 (0.55–0.84)	<0.001
Duration of hypertension	1.31 (1.00–1.70)	0.047
Maternal history of hypertension	0.72 (0.58–0.91)	0.006
Paternal history of hypertension	1.56 (1.20–2.05)	0.001
Heart failure	10.62 (6.99–16.13)	<0.001
Chronic kidney disease	1.32 (0.58–2.98)	0.510

LVH: left ventricular hypertrophy, Model fit *p*=0.010, Cox and Snell *R* Square of 0.129, Nagelkerke *R* Square of 0.172, Hosmer and Lemeshow Test of 0.721.

## Data Availability

The datasets used and analyzed during the current study are available from the corresponding author upon request.
